# Modular inhibitory coding in binary networks

**DOI:** 10.3389/fnetp.2026.1792463

**Published:** 2026-07-16

**Authors:** Bofang Wang, Michal Zochowski

**Affiliations:** 1 Department of Physics, University of Michigan, Ann Arbor, MI, United States; 2 Biophysics Program, University of Michigan, Ann Arbor, MI, United States

**Keywords:** binary network coding, binary network structure, memory capacity, memory storage, modular inhibition

## Abstract

**Introduction:**

We characterized properties of class of binary models where, as observed in biological networks, excitatory neurons are structurally and functionally separated from inhibitory units. We investigate the respective roles the two populations play in memory storage.

**Methods:**

The network is composed of separated excitatory and inhibitory layer. New patterns, represented as activation and inactivation of binary units in excitatory layer, are stored in the network through recruitment and training of inhibitory units that are grouped into individual modules and interact with excitatory layer. At the same time, the inhibitory modules compete for activation based on the signal magnitude they receive from the excitatory layer.

**Results:**

We show that inhibitory layer plays a critical role in memory storage and management, and that capacity of the network scales proportionally to number of inhibitory neurons. Further, we demonstrate that performance of the network is only gradually diminished when excitatory‐to‐excitatory (E‐E) connections are removed but critically depends on inhibitory‐to‐excitatory (I‐E) connections. We further show advantages of so designed coding scheme in terms of memory capacity, its expansion with progressive storage of new memories as well as network behavior for large memory loading.

**Discussion:**

These results are in line with new experimental work showing that inhibitory interneurons are playing critical role in memory storage and recall in the brain networks and may also address why generally excitatory networks exhibit sparser reciprocal connectivity as compared to connections to/from inhibitory units.

## 1 Introduction

Binary attractor neural networks (ANNs) have played a critical role in understanding possible dynamical processes underlying essential brain functions, such as information storage and retrieval (Hopfield, 1982; Amit, 1989; Amari, 1967). They also underlie the artificial intelligence (AI) revolution currently taking place (Raiaan et al., 2024; Matsuo et al., 2022; LeCun et al., 2015; Bengio et al., 2021). Although a number of different variants of these networks have been developed, one of the characteristics has remained prevalent—contrary to known biology, the units in the network often do not explicitly obey Dale’s law, with mixed excitatory and inhibitory connections stemming from the same units and forming mixed network layers, or inhibition is included implicitly in changes to the excitability or threshold of excitatory units (Amit, 1989; Rumelhart et al., 1986; McClelland and Rumelhart, 1988; Chauvin and Rumelhart, 1995; Palm, 1981; Palm, 1980; Knoblauch et al., 2010). Furthermore, the learning phase (connectivity dynamics) in these models is customarily separated from the recall phase (unit dynamics), preventing the storage of new patterns as information arrives. These learning rules often result in catastrophic forgetting, whereby the network loses the ability to recall any stored pattern once it becomes saturated.

Biological neuronal networks are typically differentiated into excitatory and inhibitory populations (Kandel et al., 2021), allowing for the differential modulation of network dynamics through targeted neuromodulatory inputs (Zhou et al., 2020). These two populations differ in very fundamental properties. Generally, the excitatory population is significantly larger than the number of inhibitory neurons (with the ratio varying depending on the brain modality between roughly 3:1 and 10:1), with some specialized locations (e.g., striatum and olfactory bulb) having much higher inhibitory density (Fröhlich, 2016; Bjerke et al., 2021; Benes and Berretta, 2001; Marin, 2025). Interestingly, in biological networks, excitatory neurons are generally sparsely and reciprocally connected, often forming long-range connections that target neurons in different brain modalities (Waydo et al., 2006; Braitenberg and Schüz, 1991). At the same time, the connections originating from inhibitory interneurons form dense local synaptic arborizations, with very rare long-distance connectivity. Depending on the interneuron subtype, inhibitory interneurons can target from a couple of hundred (PV + basket cells) to 1,200–1,300 (chandelier cells) pyramidal cells with robust arborizations that can silence their activity (Kubota and Kawaguchi, 2000; Znamenskiy et al., 2024). Furthermore, in the neocortex and hippocampus, excitatory pyramidal cells can sustain a firing rate of up to 50 Hz, but on average fire with a frequency below 10 Hz (Kandel et al., 2021; Dowling, 2001), whereas inhibitory interneurons encompass many subtypes, but the most numerous groups, parvalbumin-positive neurons, can support a sustained firing frequency of 100 Hz or higher (Kandel et al., 2021). This leads to a high level of divergence, where a small population of inhibitory interneurons can effectively regulate the activity of a much larger excitatory population (Booker and Vida, 2018).

Although it has been assumed that both populations are important for memory coding (Josselyn and Frankland, 2018), it was often thought that excitatory neurons carry information content, while inhibitory interneurons play a more modulatory role. Recent experimental results, however, have indicated that diverse inhibitory cell populations play a critical role in the modulation of brain dynamics and function (Raven and Aton, 2021). The interaction of these different classes of neurons is now considered critical for memory storage and recall. Furthermore, their relative excitability is thought to play a critical role in information processing (Raven and Aton, 2021; Lucas and Clem, 2018).

Here, we construct a binary network model that shares properties with the inhibitory Willshaw network (Palm, 1981; Palm, 1980; Knoblauch et al., 2010; Willshaw et al., 1969; Maltenfort et al., 1998; Golomb et al., 1990; Knoblauch, 2011; Tyrrell and Willshaw, 1992) and that separates inhibitory and excitatory neurons into two layers, with the memory representations effectively stored within the patterns of reciprocal inhibitory–excitatory (E–I and I–E) connections. Although the excitatory layer plays an input/output role, the inhibitory layer has a modular structure that allows for the constant expansion and/or redefinition of the stored memory representations through the addition, consolidation, or dynamic reassignment of inhibitory modules. This, in turn, helps mitigate the critical issue of catastrophic forgetting at increasing memory loads, when the network dynamics undergoes a phase transition that renders all stored memories unstable (Shen et al., 2023; Golden et al., 2022).

We characterize the network properties in terms of storage and retrieval performance under full connectivity and as progressively larger numbers of connections are removed. We show that the capacity of the network increases in direct proportion to the number of inhibitory cells and that although the sparsity of E–E connections only slowly degrades memory performance, reciprocal inhibitory connectivity is critical for memory storage and recall.

## 2 Methods

### 2.1 Network dynamics

**FIGURE 1:**
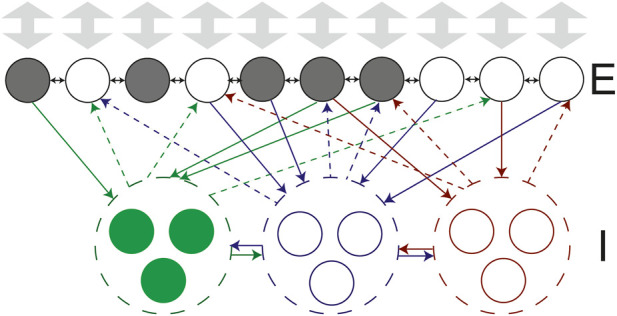
Structure of the modular excitatory/inhibitory binary network. The excitatory layer (E) is, at the same time, an input/output layer (gray arrows). The inhibitory modules (I) (dashed circles; here composed of three inhibitory neurons each) form as new representations are stored. Neural representations are composed of $\sigma_{j}=\left\{0,1\right\}$ and impinged on the excitatory layer. The excitatory units generate the field, $h_{i}^{ei}$ , which activates associated inhibitory units that form modules. These modules, on the one hand, compete with other inhibitory modules for activation (Equation 4), while on the other hand, they control the activation patterns of the excitatory layer. The inhibitory module with the largest field dominated the network dynamics, imposing its activation pattern on the excitatory layer. This leads to rapid retrieval of the representation associated with the inhibitory module.

The network is composed of separate excitatory (E) and inhibitory layers (I) (see Figure 1). The uniform excitatory layer consists of $N_{e}$ units, whose elements simultaneously serve as the inputs and outputs of the network. The inhibitory layer is composed of modules, which are clusters of inhibitory units associated with the specific memory representations. The modules are created as new binary representations are stored in the network and are composed of one or more inhibitory units.

For the excitatory units, following standard approaches (Amit, 1989), we define a signal, $h_{i}^{e}$ , that every excitatory unit receives (Equation 1):

$$h_{i}^{e}=\frac{1}{N_{e}}\sum_{j=1}^{N_{e}}J_{ij}^{e-e}\sigma_{j}^{e}-\alpha \sum_{k=1}^{N_{i}}J_{ik}^{i-e}\sigma_{k}^{i},$$ (1)

where $J_{ij}^{e-e}$ and $J_{ik}^{i-e}$ represent input connections to excitatory neurons from other excitatory (E–E connections) and inhibitory units (i.e., I–E connections), respectively; and $\alpha =\left[0,1\right]$ is the inhibitory connection multiplier, which can modulate the relative strength of the feedback inhibition controlling sparseness of the excitatory representation. Here, the network dynamics is inhibition-dominated, where effectively any neuron can control the activation/deactivation of excitatory neurons ( α=1 ).

The excitatory units, similarly to other binary network models, take two states, $\sigma_{i}^{e}=\left\{0,1\right\}$ , based on the sign local field, $h_{i}^{e}$ impinging on them (Equation 2):

$$\sigma_{i}^{e}\left(t+1\right)=\left\{\begin{array}{cc} 0 & if\;h_{i}^{e}\left(t\right)\leq 0\\ 1 & if\;h_{i}^{e}\left(t\right)>0 \end{array}\right..$$ (2)

Conversely, the inhibitory layer is composed of modules, *M*, which receive the selective input from the excitatory layer, while their outputs target other modules in the inhibitory layer, as well as units in the excitatory layer (Figure 1). The inhibitory neurons take on values $\sigma_{j}^{i}=\left\{0,1\right\}$ , with all connections originating from these neurons being negative.

The inhibitory units within the same module are not connected (i.e., they do not share inhibitory synapses), whereas all inhibitory units belonging to different modules are fully connected, creating strong lateral inhibitory interactions between units in different inhibitory modules. Here, the dynamics follows a winner-takes-all (WTA) pattern (Barron et al., 2017; Kim and Lim, 2022), with the neurons within the modules receiving the strongest excitatory input remaining active, while the others are shut down. To that extent, similarly to the excitatory units, we define the excitatory signal received by the *i*-th inhibitory neuron as in Equation 3:

$$h_{i}^{\text{ei}}\left(t\right)=\sum_{j=1}^{N_{e}}J_{\text{ij}}^{e‐i}\sigma_{j}^{e}\left(t\right),$$ (3)

where $h_{i}^{ei}$ is the excitatory field impinging on the i-th inhibitory neuron and $J_{ij}^{e-i}$ represents the strength from excitatory to inhibitory connection.

The winner-takes-all state dynamics of the inhibitory units follows the rule shown in Equation 4:

$$\sigma_{i}^{i}\left(t+1\right)=\prod_{j,j\notin M}^{N_{i}}\Theta \left(h_{i}^{\text{ei}}\left(t\right)‐h_{j}^{\text{ei}}\left(t\right)‐\varepsilon \right),$$ (4)

where $\Theta \left(.\right)$ is the Heaviside function with $\Theta \left(x<0\right)=0$ and $\Theta \left(x\geq 0\right)=1$ ; ε is an activation threshold that controls how large the field difference must be for the neuron with the lower field to be shut down; and $N_{i}$ is the number of inhibitory units in the network. Here, we set ε=0 for inter-module inhibitory units. Consequently, the only inhibitory cells that are active at a given time are those that have the highest excitatory signal, $h_{i}^{i}$ , impinging on them. The active inhibitory cells, in turn, inactivate the units in the excitatory layer through E–I connectivity, impinging on the pattern associated with the given memory.

### 2.2 Network connectivity and memory storage

Memory representations are defined through the stable activation patterns of the units in the excitatory layer, where $\vec{\xi^{m}}={\left\{0,1\right\}}^{N_{e}}$ represents the state of the *i*th excitatory unit in the *m*th stored representation (memory). Similarly to the classical ANN, memory representations are stored in the connectivity matrix (Amit, 1989); however, the algorithm is adapted to take into account existence and modularity of the inhibitory layer.

The excitatory-to-excitatory (E–E) connections are formed according to standard Hebb’s rule (Amit, 1989), $J_{ij}^{e-e}=\frac{1}{p}\sum_{m=1}^{p}\xi_{i}^{m}\xi_{j}^{m}$ . Since $\sigma_{i}^{e}=\left\{0,1\right\}$ , this effectively means that only memory representations having both units active ( $\xi_{i\;and\;j}^{m}=1$ ) form excitatory connections (see Figure 1).

The reciprocal connections between the excitatory layer and individual inhibitory modules code the memory representation (i.e., its activation pattern) assigned to a given module (Figure 1). Specifically, all active excitatory units within the given (*m*-th) memory representation, ( $\xi_{i}^{m}=1$ ), form connections to the inhibitory units composing the *m*th cluster in the inhibitory cell layer (Figure 1), i.e., ${⋀}_{k\in m}J_{i,k}^{e-i}=\xi_{i}^{m}$ , where i denotes the *i*th excitatory unit and k denotes the k-th inhibitory unit belonging to module m.

In contrast, inhibitory-to-excitatory (I–E) connections that code the inactivation pattern for the mth memory representation ( $\left.\wedge_{i}{\;\xi }_{i}^{m}=0\right)$ are formed between inhibitory units from the mth inhibitory module and the excitatory cell layer (see Figure 1). The strength of all these I–E connections is set to 1 , i.e., ${⋀}_{k\in m}{⋀}_{i,\xi_{i}^{m}=0}\;J_{k,i}^{i-e}=1$ . Thus, $J_{k,i}^{i-e}\in \left\{0,1\right\}$ . The strength of this inhibition is modulated by α , which regulates the excitatory representation sparseness. Here, α=1 , causing inhibition to dominate the state of the excitatory neurons, meaning that any activated inhibitory unit shuts down the targeted excitatory cells, irrespective of the excitatory component of the field received by these units (see Equation 1). Such strong I–E connectivity has been found to be biologically realistic. For example, chandelier cells provide highly concentrated axo-axonic inhibition, where an individual interneuron forms “cartridges” of 20–93 synapses directly on the axon initial segment of a pyramidal cell, effectively shutting down its firing (Rudy et al., 2011; Inan and Anderson, 2014; Juarez and Martinez Cerdeno, 2022; Gallo et al., 2020).

As shown in the following section, the major advantage of such a network design is that new representations can be stored progressively in the network as new experiences arrive, without interfering with previously stored memories, thereby allowing for continuous learning. During storage of the new memory representation, additional inhibitory modules are formed in the inhibitory layer as the network is presented with a new memory representation to be stored. Physiologically, these new inhibitory units could be recruited from the pool of unassigned inhibitory cells or, alternatively, dynamically form new inhibitory modules from existing but reassigned inhibitory neurons. New connections are then formed between and within the excitatory and inhibitory layers. Thus, by default, the number of excitatory cells ( $\left.N_{e}\right)$ remains constant throughout the learning process, whereas the number of inhibitory modules and, consequently, inhibitory units ( $N_{i}$ ) increases linearly with the number of stored configurations. The proportionality constant depends on the number of units per inhibitory module. We evaluated the performance of the network for modules composed of $S_{M}=1,3,5$ inhibitory neurons to account for the likely possibility that more than one inhibitory neuron regulates a given memory pattern. Biologically, the number of inhibitory neurons participating in a given memory is much higher (Booker and Vida, 2018).

## 3 Results

We develop a binary network framework in which the elements of the network are divided into separate excitatory and inhibitory populations and adhere to Dale’s law. In this framework, excitatory neurons serve as input pattern detectors and network outputs, while the inhibitory layer is divided into modules, whose inhibitory units, together with their connectivity, are critical for pattern storage and separation.

### 3.1 Storage capacity

**FIGURE 2:**
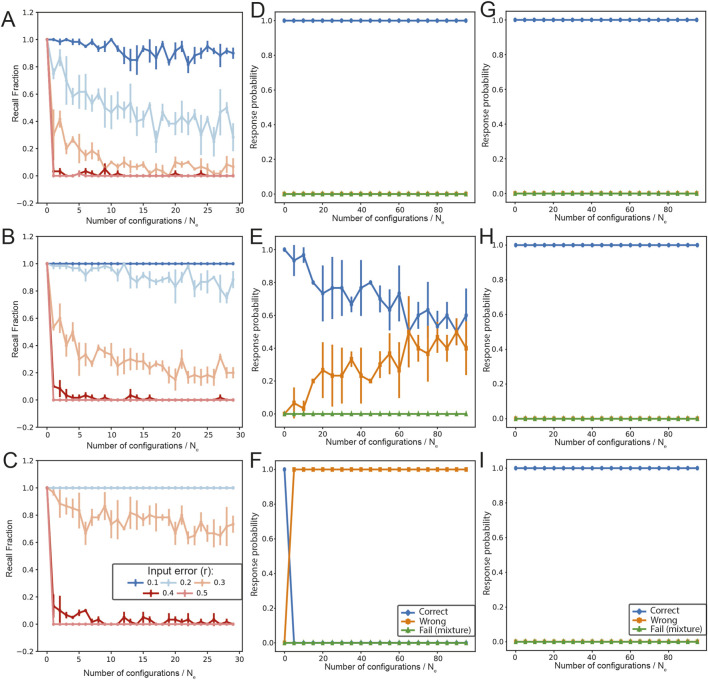
Performance of the network as a function of the onset input error for different network sizes. Performance is measured as a recall fraction (number of correctly recorded configurations/total number of recalls). Size of the network (number of excitatory neurons): **(A)** N_e_ = 50, **(B)** N_e_ = 100, and **(C)** N_e_ = 200. **(D–F)** Type of recall errors as a function of the onset input error for the network of N_e_ = 100. Here, only the ID of the initial memory was used to identify the response type. Magnitude of the input error is as follows: **(D)** input error, r = 0.1, **(E)** input error r = 0.3, and **(F)** input error, r = 0.5. Independent of the input error, the network response varies only between correct and incorrect memory identification, while the failed state is never achieved. **(G–I)** Type of recall errors corrected for the ID of the stored representation, $\xi^{\mu }$ , having the closest Hamming distance to the initial network state, $\sigma^{E}\left(t=0\right)$ , defined as $H\left(\xi^{\mu },\sigma^{E}\left(t=0\right)\right)=\sum_{k=1}^{N_{e}}\left|\xi_{k}^{\mu }-\sigma_{i}^{E}\left(t=0\right)\right|$ . As before, the magnitude of the input error is (G) input error, r = 0.1, (H) input error, r = 0.3, and (I) input error, r = 0.5. The memory with the smallest Hamming distance to the initial network state is always identified. For all simulations, the inhibitory modules consisted of a single unit. During recall error, the network converges toward different configurations rather than toward a mixed state.

We first investigated the retrieval performance of the network as a function of the number of the excitatory units, $N_{e}$ . We simulated networks with N_e_ = 50, 100, and 200 excitatory units (Figure 2). The inhibitory modules consisted of a single unit and were progressively added as the number of stored binary representations (i.e., memories) increased. The memories were defined as randomly generated N_e_ dimensional vectors of $\xi_{i}^{m}=\left\{0,1\right\}$ . The performance test consisted of retrieval of the given memory set, with progressively larger input errors, i.e., a fraction (*r = 0.1*, *0.2*, *0.3*, *0.4*, *and 0.5*) of excitatory units, $\sigma_{i}^{e}\left(t=0\right)$ , flipped to their opposite state. Larger input errors often resulted in spurious overlap with memories other than the one being tested, with the overlap for the spurious memory exceeding that of the intended memory. The memory was successfully retrieved when the overlap of the excitatory units in the final network state with the given memory $o^{m}=\frac{1}{N_{e}}\sum_{i=1}^{N_{e}}\sigma_{i}^{e}\xi_{i}^{m}>0.90$ . We measured the recall fraction, defined as the fraction of stored representations recalled correctly, by assigning a value of 1 if the memory was recalled and 0 otherwise. Each point on the panels of Figure 2 is an average of 20 random memory retrieval attempts.

We observed that the network does not saturate and fail, even when the number of stored configurations is large relative to the number of excitatory units ( $N_{e}$ ). For all network sizes (in terms of the number of units in the excitatory layer), we loaded the networks with up to $p=30\cdot N_{e}$ memories, which results in 1,500, 3,000, and 6,000 configurations stored for N_e_ = 50, 100, and 200 units, respectively. The size of the inhibitory modules was set to 1, resulting in the number of inhibitory units matching the number of stored configurations (i.e., reaching maximum values of N_i_ = 1,500, 3,000, and 6,000, respectively). We have tried larger memory loads and observed stabilization of network performance. We expect, however, that the performance of the network will start to deteriorate when the average Hamming distance, $H\left(\xi^{i},\xi^{j}\right)=\sum_{k=1}^{N_{e}}\left|\xi_{k}^{i}-\xi_{k}^{j}\right|$ (where $\xi_{k}^{i}$ is the k-th bit of the $\xi^{i}$ th memory vector) (Amit, 1989), between the stored representations within the excitatory layer becomes small (and thus overlap between the memories becomes large) so that different inhibitory modules are randomly co-activated due to the small and random field ( $h_{i}^{ei}$ ) differences that impinge on the inhibitory units.

The performance of the network deteriorates as a function of the input error (Figures 2A–C); however, it also strongly depends on the number of excitatory units in the network, $N_{e}$ . The input error of r = 0.2 results in a reduction in performance of the network to approximately 50% for the highest number of memories stored for the smallest network size with N_e_ = 50 excitatory units (Figure 2A); the performance increases to approximately 70% for N_e_ = 100 (Figure 2B). At the same time, an input error of r = 0.3 reduced network performance to only 70% for the network with N_e_ = 200 (Figure 2C).

This, however, is again explained by the increasing overlap (decreasing Hamming distance) among the memory representations in the excitatory layer for the smaller networks and, hence, by changes in the mean field magnitude, $\bar{h^{ei}}$ , impinging on the inhibitory cells from the excitatory layer. The latter is critical for the activation of the correct inhibitory module associated with the given memory (Equation 4).

We, therefore, first investigated which types of performance errors dominate during the retrieval process (Figures 2D–I). In other words, we were interested in whether, when the network failed to converge to the correct memory associated with the given initial state, it converged to another stored representation or instead settled into a mixed state with reduced overlap with any of the stored memories. To achieve this, we measured the overlap of the final network state (after the network performed a prescribed number of updates) with each stored representation. We divided network responses into three categories: correct—when the network converges to the correct memory, $\xi^{i}$ , associated with the initial network state; wrong—when the network state converges to another stored representation, $\xi^{j\neq i}$ , and fail—when none of the memories are retrieved (i.e., overlap with all the stored representations remains below the recognition threshold). We observed that, irrespective of the applied configuration error (Figures 2D–F), the network achieved only two states: correct recognition or incorrect convergence (Figure 2F). At the same time, as expected, the frequency of incorrect responses increased with the initial magnitude of the input error. The network converges to the correct memory state when r = 0.1 (Figure 2D), shows progressively worsened performance for r = 0.3 (Figure 2E), and finally fails for all memory loadings for r = 0.5 (Figure 2F).

To further characterize the performance of the network, we measured the initial overlap of the starting state with every stored memory to identify situations in which the initial Hamming distance was smaller for a memory other than the perturbed one (Figures 2G–I). We observed that, irrespective of the magnitude of the input error (r = 0.1, Figure 2G; r = 0.3, Figure 2H, and r = 0.5, Figure 2I), the network invariably converged to the memory with the smallest initial Hamming distance to the starting state, demonstrating the robustness of the network.

**FIGURE 3:**
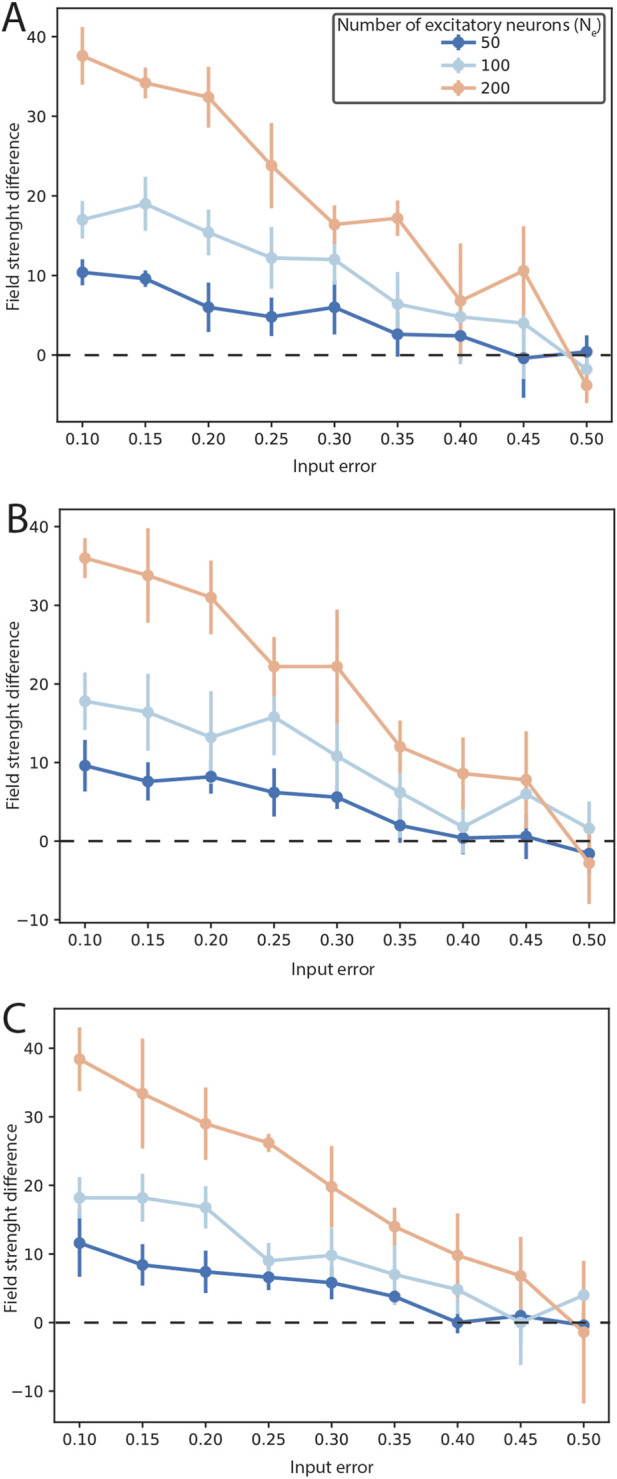
Excitatory field strength difference for various network sizes and as a function of onset input error (fraction of excitatory neurons with reversed state from stored memory). The ratio of stored configurations to the number of excitatory neurons, $P_{E}=\frac{p}{N_{e}}$ , is as follows: **(A)** *P*_*E*_ = 5, **(B)** *P*_*E*_ = 50, and **(C)** *P*_*E*_ = 100. Networks with a higher number of excitatory neurons, N_e_, have larger field separation for the same network loading, allowing for better memory recall.

This result is because the network dynamics is dominated by a winner-takes-all effect generated by the inhibitory modules. This effect is driven by the relative magnitude of the excitatory field arriving at inhibitory units within the module. We, therefore, investigated how the field depends on excitatory network size, memory loading (i.e., number of stored configurations), and input error (Figure 3). We observed that the mean excitatory field difference between random configuration and the representation stored in the network does not show strong dependence on memory loading of the excitatory layer (i.e., $P_{E}=\frac{p}{N_{e}}$ ; *P*_*E*_ = 5, Figure 3A; *P*_*E*_ = 50, Figure 3B; *P*_*E*_ = 100, Figure 3C). However, it critically depends on the excitatory network size and input error (Figures 3A–C, x-axis). Since the selective activation of inhibitory modules, and hence correct memory recall, depends on the separation of the excitatory fields of activated representation from those of all other stored memories, this separation plays a critical role in memory retrieval. Thus, the size of the excitatory layer population N_e_ is critical for reducing the correlation among stored memory representations.

### 3.2 Network performance upon the removal of connections

**FIGURE 4:**
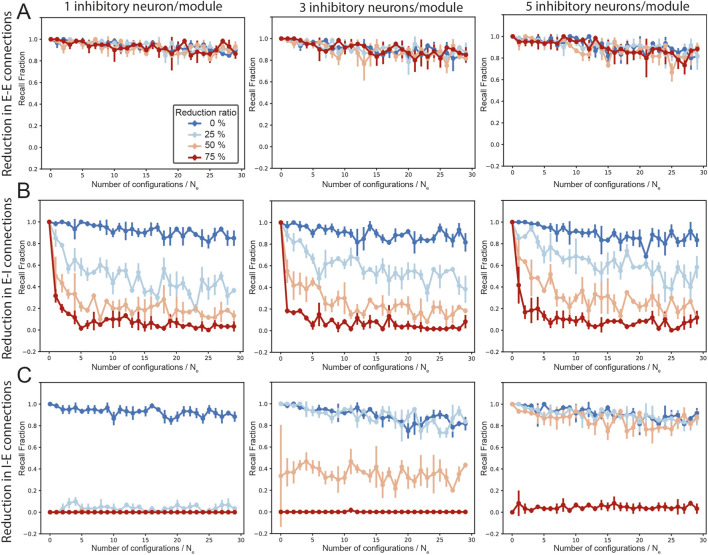
Reduction in network performance (measured as recall fraction) as a function of sparsity of connections for different number of inhibitory units in inhibitory modules. The inhibitory modules consisted of one (left column), three (center column), and five inhibitory neurons (right column). In all panels, the number of connections was reduced (random removal) by 0%, i.e., full connectivity (dark blue line), 25% (light blue line), 50% (orange line), and 75% (red line). Rows: **(A)** removal of E–E connections, **(B)** E–I connections, and **(C)** I–E connections. Network performance is robust to the removal of E–E connections irrespective of the number of inhibitory neurons/module, and is most sensitive to the removal of I–E connections. The increase in the number of inhibitory neurons per module enhances performance.

**FIGURE 5:**
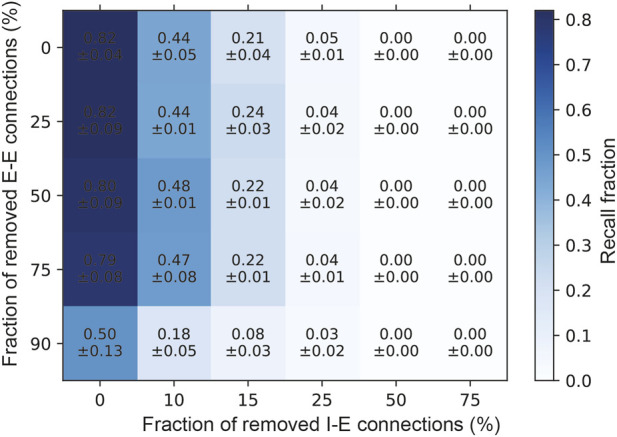
Network performance as a function of the fraction of connections removed. The network composed of N_e_ = 50 with 150 stored configurations is tested, with inhibitory modules consisting of one inhibitory unit. The results highlight a much stronger dependence of network performance on the number of I–E connections removed than on the number of missing E–E connections.

Next, we characterized network performance as network connections were progressively removed (Figures 4, 5). We separately removed E–E, E–I, or I–E connections and measured network recall performance (defined as above) for different numbers of units in the inhibitory modules. We evaluated network performance for $S_{M}=1,3,5$ inhibitory units per module.

We observed that the network recall performance was extremely robust to the removal of E–E connections (Figure 4A) across any number of inhibitory units per module, $S_{M}=1,3,5$ . Only the removal of ∼90% of connections (Figure 5) resulted in a significant difference in recall statistics.

On the other hand, random removal of E–I connections made a significant impact on network recall as it interferes with the inhibitory neurons’ mean field $\bar{h^{ei}}$ , and we observed a precipitous decrease in performance for the increasing fraction of removed connections (Figure 4B; left column). This effect was partially offset by increasing the number of units in inhibitory modules (Figure 4B; center and right columns). The inclusion of five units ( $S_{M}=5$ ) per inhibitory module increased recall performance by 10%–20% for every connection removal fraction (Figure 4B, right column). This effect is driven by the fact that improved statistics of inhibitory field sampling (due to multiple inhibitory units in a module) could lead to partial recovery of baseline performance.

Finally, the strongest effect of connection removal was observed in the case of the removal of I–E connections (Figure 4C). Here, elimination of even a low fraction of connections in a network composed of inhibitory modules consisting of a single unit resulted in a precipitous decrease in network performance (Figure 4C, left column). This effect is driven by the fact that I–E connections are solely responsible for impinging the correct unit inactivation pattern on the excitatory layer, associated with the recall of a given memory. Conversely, missing I–E connections leave spurious excitatory units activated.

Here, the increasing number of units within each inhibitory module dramatically improves network recall performance by providing robustness through the targeting of the correct excitatory neurons by multiple units within individual inhibitory modules (Figure 4C; center and right panels).

### 3.3 Network performance in the presence of noise

**FIGURE 6:**
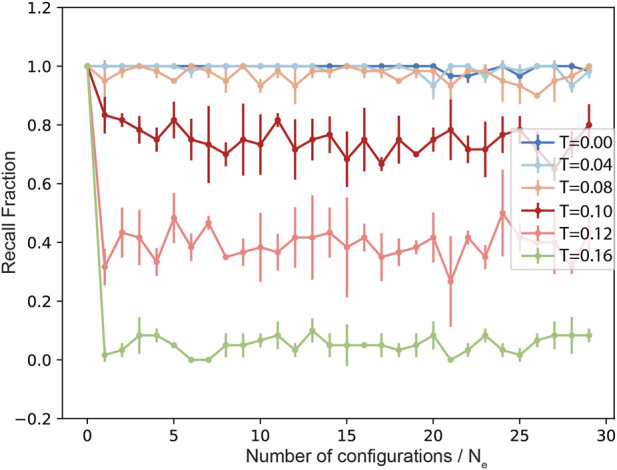
Network performance as a function of the noise level (T). The network is composed of N_e_ = 100 excitatory neurons, with five inhibitory neurons per inhibitory module. Network performance in the presence of noise does not depend on network loading.

Finally, we investigated how the network performs in the presence of noise (Figure 6). Following standard approaches (Amit, 1989), the noise is defined as a reduced probability of a unit achieving a state determined by the local field it receives (Equation 5):

$$\mathrm{Pr}\left\{\sigma_{i}^{e}\left(t+\Delta t\right)\left.=1\right|h_{i}^{\text{e}}\left(t\right)\right\}=0.5\left[1+\tanh\left(\frac{1}{T}h_{i}^{\text{e}}\left(t\right)\right)\right],$$ (5)

where $\left.T\in [0,+\infty \right)$ is a noise parameter, with zero denoting noise-free deterministic dynamics.

Here, we added noise only to the excitatory layer, with the underlying physiological reason being that excitatory neurons are more prone to noise dynamics because they are directly associated with noisy input. Additionally, in the brain, reciprocal E–E connectivity is sparse. Inhibitory neurons, on the other hand, receive more convergent input and form denser arborizations (Hofer et al., 2011), likely leading to noise cancellation. Adding noise to the inhibitory cells should not change qualitatively obtained results.

We measured how network recall performance changes as a function of memory loading in the presence of noise. We varied the noise level between $T\in \left[0,0.16\right]$ . We observed (Figure 6), as expected, that network performance decreases as the noise level increases, but it is largely independent of memory loading (it quickly asymptotes to a constant value for larger memory loading). This is due to the independence of inhibitory modules mediating specific memory recall.

## 4 Discussion and conclusions

In this manuscript, we describe a binary neural network framework that separates excitatory and inhibitory signaling. We argue that, in addition to being more physiologically realistic, the proposed framework has multiple performance advantages over standard Hopfield-type binary attractor neural networks (Hopfield, 1982).

The proposed framework shares common features with the so-called inhibitory Willshaw network (Knoblauch et al., 2010; Willshaw et al., 1969; Golomb et al., 1990; Knoblauch, 2011; Tyrrell and Willshaw, 1992; Knoblauch and Palm, 2001). Here, the excitatory layer carries the overall network excitation and plays an important input/output role, while information storage and recall are critically controlled by the inhibitory network layer. The inhibitory layer is divided into modules that separately mediate activation/deactivation of specific excitatory units, leading to recall of the associated memory. The inhibitory units belonging to different modules follow winner-takes-all dynamics, allowing for the recall of the memory most closely associated with the input while suppressing spurious contributions from other inhibitory modules. Although this is an idealized representation, a similar modular functional network structure was observed in biological networks (Sporns and Betzel, 2016; Mulholland et al., 2021) and is thought to underlie inhibitory control (Spielberg et al., 2015).

The excitatory representations constituting memories are iteratively stored through the recruitment and formation of new inhibitory modules. This makes the capacity of the network linearly proportional to the number of modules (and thus to the number of inhibitory units in the network). Analogously to the results presented here, experimental data analysis and modeling have shown that inhibitory connectivity, rather than excitatory connectivity, possibly controls the information maintenance in the cortex (Mongillo et al., 2018) and that the number of inhibitory neurons in the network is directly related to the maximal number of neural assemblies that can be consolidated, indicating that inhibition has a direct impact on the memory capacity of the neural network (Bergoin et al., 2023).

Furthermore, the formation of inhibitory modules can be considered the emergence of inhibitory engram neurons. Such engrams have been observed in biological networks and, as here, are critical for memory selectivity (Barron et al., 2017; Tome et al., 2024; Hou et al., 2024).

Finally, the presented coding scheme has several additional advantages. We have shown that the network can store new configurations progressively as they arrive, independent of its earlier history, limiting the interference between the already stored memory representations. Furthermore, because of the independence of the module connectivity, the network never experiences catastrophic forgetting (Shen et al., 2023; Golden et al., 2022) associated with overloading, which is a standard problem for other networks of similar type. At the same time, the network can be extended to include gradual forgetting of less frequently accessed memories by progressively weakening excitatory connections to the associated inhibitory modules.

We further show that, although network performance is robust to the removal of E–E connections, E–I connectivity, and, even more importantly, I–E connectivity play critical roles in memory recall. These properties closely follow the structure of the biological brain networks, where it has been shown that the reciprocal (E–E) connectivity between excitatory neurons in the brain is generally sparse, whereas both E–I connections and feedback I–E connections form dense connectivity trees (Hofer et al., 2011). At the same time, I–I connectivity forms dense structures directly on the same interneuron types or indirectly acting through other interneurons (Swanson and Maffei, 2019; Raven and Aton, 2021; Fino et al., 2013).

**FIGURE 7:**
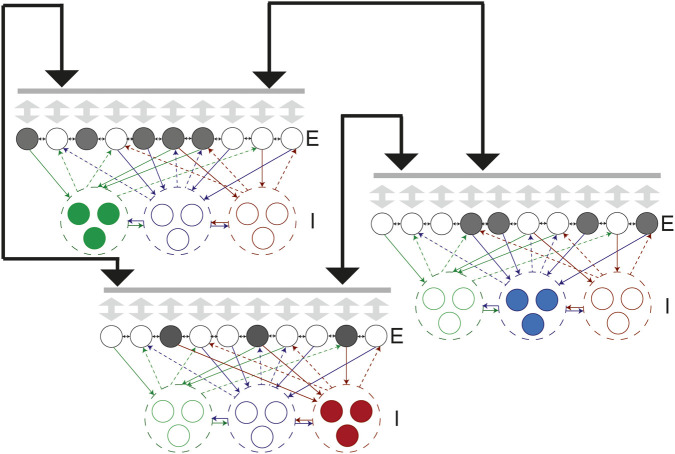
Modular network design. The memory representation is composed of multiple features. The features are stored locally via modular inhibition, whereas the excitatory connectivity links the features together across network modules.

These results lead to several constraints/predictions regarding the structure of biological networks. First, in the proposed model, the number of inhibitory neurons required to store a given memory set scales with the number of memories to be stored. In biological networks, the size of the population of inhibitory cells is consistently between 10% and 30% of that of their excitatory counterparts, with different brain modalities having somewhat different ratios; for example, the human hippocampus has an average I–E cell ratio of only 10%–15%, whereas the human neocortex has an I–E ratio of approximately 33% (Le Magueresse and Monyer, 2013). Since the storage capacity in the hippocampus is relatively low compared to that in the cortex, this density difference could potentially be explained by differences in the needed storage capacity. Furthermore, the density of the inhibitory interneurons has been found to be lower in the rodent neocortex (10%–15%) than in the human neocortex (∼33%) (Marin, 2025; Pelkey et al., 2017). This fact could further point to the need for an increased storage capacity and a potential increase in the complexity of human memories. At the same time, inhibitory interneurons need to form dense incoming and outgoing connectivity trees with the corresponding excitatory pyramidal cells. Parvalbumin-positive (PV+) chandelier cells form dense local networks, targeting the axon initial segments of pyramidal cells with axonal arbors that provide powerful, fast-spiking control over their excitatory counterparts (Inan and Anderson, 2014; Juarez and Martinez Cerdeno, 2022; Gallo et al., 2020). These local inhibitory networks are very dense, with the connectivity probabilities reaching 70% within the 200 µm radius, leading to extremely tight control of excitatory activity patterns (Znamenskiy et al., 2024; Buzsaki et al., 2007). At the same time, their total number of excitatory and inhibitory inputs is estimated to be tens of thousands of synapses (Druga et al., 2023). In contrast, excitatory cells have significantly sparser connectivity, with a larger fraction of their connections being long-range rather than local [cite]. This leads to the possibility that the cortical network is modular, a notion that has been experimentally supported (Rao-Ruiz et al., 2019), with local networks storing representations of specific memory features through local inhibitory interneuron circuits and excitatory neurons binding these local representations together into complete memories (Figure 7). This would reduce the need for an exorbitant number of inhibitory interneurons and their extensive arborization trees.

Finally, for the inhibitory interneurons to play a key role in the formation of memory engrams, their connectivity must exhibit a high degree of plasticity. It has been found experimentally that inhibitory synaptic plasticity (ISP) can exhibit a significantly faster rate, greater learning magnitude, or stronger overall network dominance than excitatory synaptic plasticity (ESP) (Znamenskiy et al., 2024; Miehl and Gjorgjieva, 2022; Gravielle et al., 2022).

At the same time, the model represents an idealization and has a number of drawbacks compared with other models of this type (Palm, 1981; Palm, 1980; Knoblauch et al., 2010; Golomb et al., 1990; Knoblauch, 2011; Knoblauch, 2007). Since the network stores random global binary patterns, the inhibitory-to-excitatory connectivity scales with ∼ N_e_. This leads to a lower information density bit/synapse than the optimal value (Knoblauch et al., 2010; Golomb et al., 1990; Knoblauch, 2011; Knoblauch, 2007). This drawback can be partially mitigated by modular design, as presented in Figure 7. Here, the inhibitory interneurons store/separate only local distinct memory features, which are later bound together via long-range excitation.

Another issue is the scaling of inhibitory neurons with the number of stored representations. Although this may lead to an exorbitant number of inhibitory neurons, it remains unclear what the ratio of the number of information bits stored within individual representations to the number of representations stored (and, hence, the number of inhibitory interneurons) in biological neural networks is, and if it is <1, this may not pose an issue.

Overall, the advantages of the presented binary network framework should be of interest to both the neuroscience and AI communities.

## Data Availability

The raw data supporting the conclusions of this article will be made available by the authors, without undue reservation.
